# Higher allostatic load at hospitalization is linked to worse outcome 90 days after stroke

**DOI:** 10.3389/fstro.2026.1847419

**Published:** 2026-08-14

**Authors:** Eva Birgitte Aamodt, Karim Borei, Thomas Potter, Farhaan Vahidy

**Affiliations:** 1 Division of Radiology and Nuclear Medicine, Oslo University Hospital, Oslo, Norway; 2 Department of Neurosurgery, Houston Methodist, Houston, TX, United States; 3 Center for Health Outcomes and Informatics Research in Rehabilitation (CHOIRR), TIRR Memorial Hermann, Houston, TX, United States

**Keywords:** allostatic load, dependency, length of stay, mortality, stress, stroke

## Abstract

**Background:**

Allostatic load (AL), a marker of cumulative stress-related physiologic burden, may influence acute ischemic stroke (AIS) outcome through multisystem dysregulation.

**Methods:**

A total of 4,103 AIS patients were recruited from Houston Methodist Registry of Neurological Endpoint Assessments among Patients with Ischemic and Hemorrhagic Stroke (REINAH). Clinical information was collected at initial hospital stay and AL was created using ten parameters representing the metabolic, inflammatory and vascular systems. No neuroendocrine parameters were available. Length of stay (LOS) at the hospital was collected upon departure from the initial hospitalization, and mortality and dependency (modified Rankin Scale) were collected at 90 days post stroke. Factors independently associated with high AL were examined using a multivariable logistic regression model. Associations between baseline AL and post-stroke outcomes (LOS, mortality, and dependency) were examined using unadjusted and adjusted regression models.

**Results:**

High AL was associated with longer LOS, and higher mortality and dependency at 90 days. Factors independently associated with high AL included younger age, Black race, higher area deprivation index, being single, illegal drug use, diabetes mellitus, sepsis, higher Charlson comorbidity index, anticoagulation reversal therapy, antihypertensive therapy, and higher NIHSS at 6 and 24 h post stroke, whereas being a woman, hypercholesterolemia, atrial fibrillation, anticoagulant therapy, intravenous tissue plasminogen activator treatment, and endovascular thrombectomy were associated with moderate-low AL.

**Conclusion:**

High AL is associated with poorer functional and clinical outcomes after AIS and may support future risk stratification work.

## 1 Introduction

Stroke is the third leading cause of death and disability worldwide (World Health Organization, 2024), with 90% of strokes being linked to modifiable risk factors such as hypertension, physical inactivity, smoking, heart disease, depression, and chronic stress (World Stroke Organization, 2025). Chronic stress increases the risk of hypertension, atherosclerosis, coagulability, and adverse health behaviors, partly through sustained neuroendocrine and autonomic activation that produces cumulative physiologic strain. Over time, this “wear and tear” promotes inflammatory and vascular dysregulation, leaving the body and brain more vulnerable to injury (Guidi et al., 2021) and potentially thereby also worsening the effects of a stroke.

One way to quantify this long-term physiologic burden is through allostatic load (AL), which captures the cumulative impact of chronic stress on metabolic, inflammatory, vascular, and neuroendocrine systems (McEwen and Stellar, 1993). There is no gold standard for how to measure AL, but including markers from all four physiological systems is considered optimal for capturing its full complexity (Whelan et al., 2021). Dysregulation across these systems overlaps strongly with pathways implicated in stroke risk and recovery, including chronic low-grade inflammation, endothelial dysfunction, and impaired vascular reactivity (Benjamin et al., 2019; Cacciatore et al., 2025; Cercel et al., 2025).

AL has been linked to many diseases and to several established risk factors for poor stroke outcome (Evans et al., 2025), yet relatively little work has examined AL directly in the context of stroke itself (Jenkinson et al., 2026). The aim of this study is to describe characteristics associated with high AL in acute ischemic stroke (AIS) patients and to evaluate its relationship with in-hospital and long-term outcomes. We hypothesized that higher AL would be associated with longer stay at hospital in relation to the stroke, as well as higher 90-day post-stroke mortality and dependency.

## 2 Material and methods

### 2.1 Sample and inclusion criteria

Patients with AIS were included from Houston Methodist Registry of Neurological Endpoint Assessments among Patients with Ischemic and Hemorrhagic Stroke (REINAH), with a total of *N* = 4,103, among which 2,244 (54.7%) had available 90-day modified Rankin Scale (mRS) assessment.

REINAH is an observational registry of patients admitted to the Houston Methodist hospital system which comprises seven certified stroke centers in and around Houston, Texas, USA. Drawing from electronic health records, the REINAH system automatically collects and cleans data from all adult patients (≥ 18 years) admitted with primary non-traumatic stroke, identified using encounter-based ICD-10 codes. Patient computed tomography (CT) and magentic resonance imaging (MRI) images collected during primary hospitalization are retained within REINAH, as are patient and encounter characteristics. Outcomes for patients with intracerebral hemorrhage are collected via phone at 30, 90, 180, and 365 days after discharge, with additional records for AIS patients provided by the Hospital Outcomes-based Prospective Endpoints in Stroke (HOPES), which collects 90-day functional outcomes using the mRS.

The REINAH study protocol was approved by the Houston Methodist Institutional Review Board (IRB; Approval No. PR00025034). All procedures were performed in accordance with the ethical standards of the institutional research committee and with the 1964 Declaration of Helsinki and its later amendments. Written informed consent was obtained from all individual participants included in this study.

### 2.2 Allostatic load characteristics

The AL score was created using ten parameters representing different systems; *metabolic:* body-mass index (BMI), glycated hemoglobin (HbA1C), total cholesterol, triglycerides, and high-density lipoprotein (HDL). *Inflammatory:* C-reactive protein (CRP), and white blood cell count (WBC). *Vascular:* Systolic—and diastolic blood pressure (SBP and DBP), and pulse. No neuroendocrine parameters were available.

The parameters were scored by quartiles. For all parameters except HDL, numeric values were arranged from lowest to highest, then partitioned into quartiles from the lowest to the highest. For HDL lower values indicate greater risk, so the numeric values were arranged from highest to lowest and then partitioned into quartiles, whereby the highest values were assigned to the lowest quartile. A point was then ascribed per parameter if the value fell within the highest quartile. Points from all the ten parameters were then summed to create the AL score (min = 0, max = 10). A score of ≥ 4 was considered a high AL. There is no universally accepted cutoff for high AL, although a threshold of ≥3 is commonly used (Beese et al., 2022). We applied a higher cutoff due to the high burden of comorbidity in stroke populations, consistent with approaches used in prior studies (Duong et al., 2017; Geronimus et al., 2006).

### 2.3 Clinical characteristics

Demographic and clinical data were collected by study radiologists shortly after presentation with AIS, as part of standard care. Based on previous literature on risk factors for poor post stroke outcome and on risk of higher AL, the following baseline factors were included:

**Sociodemographic:** age, sex, race, state area deprivation index (ADI), marital status, tobacco use, alcohol use, and illegal drug use. Age was grouped into ≤ 64, 65–74, 75–84, and ≥ 85. Race was grouped as Asian, Black, Other, and White. Marital status was grouped as divorced/widowed, married/partner, and single.

**Comorbidities:** hypercholesterolemia, hypertension, atrial fibrillation (AF), diabetes mellitus, diabetes mellitus with complications, sepsis, Charlson comorbidity index (CCI), and medications (statin therapy, antiplatelet therapy, anticoagulant therapy, and antihypertensive therapy) were included. Hypercholesterolemia was defined as lipid-modifying treatment prior to admission and/or total cholesterol ≥ 6.2 and/or LDL ≥ 4.1. Hypertension was defined as pre-stroke use of antihypertensive therapy. AF was defined as a history of permanent or paroxysmal AF or atrial flutter detected in electrocardiogram and described in medical records and/or permanent or paroxysmal AF or atrial flutter detected in electrocardiogram and/or telemetry during hospital stay. Diabetes mellitus was defined as a history of diabetes mellitus from medical records and/or pre-stroke use of antidiabetic medication and/or HbA1c ≥ 6.5% at admittance for stroke.

**Stroke characteristics:** stroke severity measured with the National Institute of Health Stroke Scale (NIHSS), time to arrival (minutes), Intravenous Tissue Plasminogen Activator (IVTPA), and Endovascular Thrombectomy (EVT) were included. NIHSS scores at 6 and 24 h were used in the analyses. These measures were selected because admission NIHSS contained a greater proportion of missing data, allowing inclusion of a larger number of participants in the analyses. NIHSS ranges from 0 to 42, with higher scores indicating more severe strokes. NIHSS was grouped into 0 = no stroke, 1–4 = minor stroke, 5–15 = moderate stroke, 16–20 = moderate to severe stroke, and 21–42 = severe stroke.

Some of the variables had missing data. From the 4,103 of the full cohort and 2,244 of the sub-cohort: ADI was available for 4,069 in the full cohort and 2,220 in the sub-cohort. Alcohol use was available for 4,017 in the full cohort and 2,192 in the sub-cohort. Illegal drug use was available for 3,917 in the full cohort and 2,136 in the sub-cohort. Hypertension, AF, diabetes, diabetes with complications, and CCI were available for 4,059 in the full cohort and 2,241 in the sub-cohort. NIHSS as 6 h was available for 3,580 in the full cohort and 2,001 in the sub-cohort. NIHSS at 24 h was available for 3,820 in the full cohort and 2,124 in the sub-cohort. Time to arrival was available for 785 participants in both the full cohort and the sub-cohort.

### 2.4 Outcome assessment

The outcome measures included length of stay (days), mortality at 90 days post stroke (deceased/alive), and dependency as mRS at 90 days post stroke. mRS ranges from 0 to 6, with higher scores indicating increased disability (Wilson et al., 2005). Functional dependency was defined as mRS ≥ 3, including death (mRS = 6), consistent with widely used distinction between functional independence (mRS 0–2) and dependency (3–6).

### 2.5 Statistical analysis

Statistical analyses were performed using R v. 3.3.3 (R Core Team 2017) and Stata v. 16. Means and standard deviations (SD) were calculated, and outliers and normality checked using histograms.

Student *t*-tests, chi square tests, or ANOVA per cohort (full cohort + sub-cohort) were run for all factors to check for significant differences between the high AL group and the moderate-low AL group.

Associations with high AL were assessed by fitting a multivariate logistic regression model, adjusted for age-group, sex, race, ADI, marital status, tobacco use, alcohol use, and illegal drug use. Univariate and multivariate adjusted associations with the outcome variables were assessed by fitting logistic regression models (90-day mortality, 90-day dependency) and linear regression models (length of stay). Multivariable modeling was performed at different levels of control for additional covariates, including a partially adjusted model (high AL, age-group, sex, race, NIHSS-group, IVTPA, and EVT) and a fully adjusted multivariate model (high AL, age-group, sex, race, NIHSS-group, IVTPA, EVT, hypercholesterolemia, sepsis, statin therapy, antihypertensive therapy, anticoagulant therapy, anticoagulation reversal therapy, and antiplatelet therapy).

Sensitivity analyses were run for all partially adjusted models and multivariate outcome models (mortality, length of stay, and dependency). As CRP and WBC may partly reflect the acute stroke response rather than chronic physiological burden, we conducted sensitivity analyses using an alternative AL score excluding CRP and WBC (8 biomarkers; range 0–8). For this reduced score, high AL was defined as ≥ 3.

Multicollinearity was assessed for all the multivariate models using variance inflation factors (VIFs). All VIF were below 2, indicating no evidence of problematic collinearity among the covariates. VIF values varied slightly across models due to differing sample compositions.

## 3 Results

### 3.1 Baseline clinical, brain and stroke characteristics

In the full cohort (Table 1), high AL compared to moderate-low AL was associated with lower age [Mean (M): 62.23 (SD: 14.23) vs. 67.82 (15.36), respectively], Black race (36.52% vs. 30.32%), higher ADI [5.37 (2.85) vs. 4.72 (2.97)], single status (28.64% vs. 21.96%), illegal drug use (11.12% vs. 8.32%, *p* = 0.008), diabetes mellitus (32.84% vs. 22.12%, *p* < 0.001), sepsis (10.14% vs. 4.28%, *p* < 0.001), a higher CCI [5.11 (3.16) vs. 4.55 (3.15)], being on anticoagulation reversal therapy (2.66% vs. 1.55%, *p* = 0.023), antihypertensive therapy (86.42% vs. 80.14%, *p* < 0.001), and a higher NIHSS at both 6 h [7.26 (7.77) vs. 6.01 (7.08) and 24 h (7.13 (7.70) vs. 5.62 (6.80))].

**Table 1:** Baseline characteristics.

	Full cohort (L.O.S + mortality)				Sub-cohort (L.O.S + mortality + mRS)			
	Total	High AL	Moderate- low AL	*p*-value	Total	High AL	Moderate- low AL	*p*-value
***N***	4,103	1,016	3,087		2,244	538	1,706	
Sociodemographic								
Age *M* (SD)	66.44 (15.28)	62.23 (14.23)	67.82 (15.36)	**<0.001**	67.63 (15.06)	63.28 (14.17)	69.00 (15.08)	**<0.001**
Age group				**<0.001**				**<0.001**
0–64 years	1,688 (41.14%)	562 (55.31%)	1,126 (36.48%)		845 (37.66%)	287 (53.35%)	558 (32.71%)	
65–74 years	1,090 (26.57%)	258 (25.39%)	832 (26.95%)		634 (28.25%)	142 (26.39%)	492 (28.84%)	
75–84 years	854 (20.81%)	128 (12.60%)	726 (23.52%)		465 (20.72%)	61 (11.34%)	404 (23.68%)	
85 + years	471 (11.48%)	68 (6.69%)	403 (13.05%)		300 (13.37%)	48 (8.92%)	252 (14.77%)	
Women	2,084 (50.79%)	487 (47.93%)	1,597 (51.73%)	**0.036**	1,116 (49.73%)	267 (49.63%)	849 (49.77%)	0.96
Race				**0.008**				0.053
Asian	196 (4.78%)	42 (4.13%)	154 (4.99%)		95 (4.23%)	21 (3.90%)	74 (4.34%)	
Black	1,307 (31.85%)	371 (36.52%)	936 (30.32%)		729 (32.49%)	202 (37.55%)	527 (30.89%)	
Other	167 (4.07%)	38 (3.74%)	129 (4.18%)		80 (3.57%)	15 (2.79%)	65 (3.81%)	
White	2,429 (59.20%)	564 (55.51%)	1,865 (60.41%)		1,338 (59.63%)	300 (55.76%)	1,038 (60.84%)	
ADI state^m^	4.88 (2.95)	5.37 (2.85)	4.72 (2.97)	**<0.001**	4.88 (3.02)	5.39 (2.91)	4.73 (3.04)	**<0.001**
Marital status				**<0.001**				**<0.001**
Divorced/widowed	964 (23.50%)	199 (19.59%)	765 (24.78%)		561 (25.00%)	110 (20.45%)	451 (26.44%)	
Married/partner	2,082 (50.74%)	504 (49.61%)	1,578 (51.12%)		1,148 (51.16%)	268 (49.81%)	880 (51.58%)	
Single	969 (23.62%)	291 (28.64%)	678 (21.96%)		480 (21.39%)	142 (26.58%)	337 (19.75%)	
Tobacco use	1,497 (36.49%)	379 (37.30%)	1,118 (36.22%)	0.53	813 (36.23%)	195 (36.25%)	618 (36.23%)	0.99
Alcohol use^m^	1,995 (49,66%)	469 (47.09%)	1,526 (50.51%)	0.061	1,062 (48.45%)	233 (44.30%)	829 (49.76%)	**0.029**
Drug use^m^	353 (9.01%)	108 (11.12%)	245 (8.32%)	**0.008**	165 (7.72%)	46 (8.95%)	119 (7.34%)	0.23
Comorbidities								
Hypercholesterolemia	711 (17.33%)	146 (14.37%)	565 (18.30%)	**0.004**	347 (15.46%)	67 (12.45%)	280 (16.41%)	**0.027**
Hypertension^m^	2,510 (61.84%)	635 (63.00%)	1,875 (61.46%)	0.38	1,365 (60.91%)	334 (62.20%)	1,031 (60.50%)	0.48
AF^m^	996 (24.54%)	204 (20.24%)	792 (25.96%)	**<0.001**	641 (28.60%)	130 (24.21%)	511 (29.99%)	**0.010**
Diabetes m.^m^	1,006 (24.78%)	331 (32.84%)	675 (22.12%)	**<0.001**	543 (24.23%)	167 (31.10%)	376 (22.07%)	**<0.001**
Diabetes m. w compl.^m^	946 (23.31%)	331 (32.84%)	615 (20.16%)	**<0.001**	498 (22.22%)	166 (30.91%)	332 (19.48%)	**<0.001**
Sepsis	235 (5.73%)	103 (10.14%)	132 (4.28%)	**<0.001**	124 (5.53%)	53 (9.85%)	71 (4.16%)	**<0.001**
Charlson comorbidity^m^	4.68 (3.16%)	5.11 (3.16)	4.55 (3.15)	**<0.001**	4.98 (3.23)	5.59 (3.33)	4.79 (3.17)	**<0.001**
Statin therapy	3,467 (84.50%)	856 (84.25%)	2,611 (84.58%)	0.80	1,870 (83.33%)	447 (83.09%)	1,423 (83.41%)	0.86
Antiplatelet therapy	3,288 (80.14%)	798 (78.54%)	2,490 (80.66%)	0.14	1,748 (77.90%)	410 (76.21%)	1,338 (78.43%)	0.28
Anticoagulant therapy	1,163 (28.35%)	244 (24.02%)	919 (29.77%)	**<0.001**	714 (31.82%)	155 (28.81%)	559 (32.77%)	0.086
Anticoagulation reversal therapy	75 (1.83%)	27 (2.66%)	48 (1.55%)	**0.023**	44 (1.96%)	12 (2.23%)	32 (1.88%)	0.61
Antihypertensive therapy	3,352 (81.70%)	878 (86.42%)	2,474 (80.14%)	**<0.001**	1,838 (82.91%)	465 (86.43%)	1,373 (80.48%)	**0.002**
Stroke characteristics								
NIHSS—first 6 h ^m^	6.31 (7.27)	7.26 (7.77)	6.01 (7.08)	**<0.001**	7.60 (7.93)	8.88 (8.29)	7.22 (7.78)	**<0.001**
NIHSS—first 24 h ^m^	5.99 (7.06)	7.13 (7.70)	5.62 (6.80)	**<0.001**	7.17 (7.70)	8.56 (8.28)	6.73 (7.46)	**<0.001**
Time to arrival^m^ (min)	305.02 (882.93)	312.05 (692.41)	303.40 (921.72)	0.91	305.02 (882.93)	312.05 (692.41)	303.40 (921.72)	0.91
IVTPA	882 (21.50%)	172 (16.93%)	710 (23.00%)	**<0.001**	600 (26.74%)	115 (21.38%)	485 (28.43%)	**0.001**
EVT	492 (11.99%)	103 (10.14%)	389 (12.60%)	**0.036**	423 (18.85%)	87 (16.17%)	336 (19.70%)	0.068
AL measures								
BMI	28.84 (6.80)	32.08 (7.69)	27.77 (6.12)	**<0.001**	28.72 (6.79)	32.07 (7.91)	27.66 (6.03)	**<0.001**
HbA1c (%)	6.49 (1.79)	7.55 (2.34)	6.14 (1.39)	**<0.001**	6.44 (1.75)	7.49 (2.32)	6.11 (1.37)	**<0.001**
Total cholesterol (mg/dL)	166.62 (48.50)	183.74 (60.18)	160.98 (42.51)	**<0.001**	165.32 (48.08)	183.29 (59.87)	159.66 (42.16)	**<0.001**
Triglycerides (mg/dL)	129.05 (93.12)	188.08 (139.26)	109.62 (60.19)	**<0.001**	124.52 (86.54)	183.73 (128.51)	105.85 (56.52)	**<0.001**
HDL (mg/dL)	48.90 (17.38)	41.75 (17.00)	51.25 (16.86)	**<0.001**	48.94 (17.22)	41.36 (16.74)	51.33 (16.67)	**<0.001**
CRP (mg/L)	19.85 (42.42)	34.40 (59.90)	15.06 (33.46)	**<0.001**	19.17 (40.98)	34.04 (58.54)	14.49 (32.33)	**<0.001**
WBC (10^3^ cells/μL)	8.41 (4.06)	10.17 (5.69)	7.83 (3.14)	**<0.001**	8.62 (4.36)	10.39 (6.26)	8.05 (3.36)	**<0.001**
SBP first 24 h	144.73 (20.69)	153.84 (22.89)	141.74 (18.98)	**<0.001**	144.11 (20.38)	153.08 (22.74)	141.28 (18.71)	**<0.001**
DBP first 24 h	75.28 (10.70)	80.89 (11.98)	73.43 (9.55)	**<0.001**	74.60 (10.72)	80.28 (11.99)	72.81 (9.62)	**<0.001**
Pulse	76.90 (13.15)	84.23 (13.92)	74.49 (11.95)	**<0.001**	76.97 (13.11)	84.02 (13.83)	74.75 (12.05)	**<0.001**
AL score	2.16 (1.59)	4.76 (0.97)	1.64 (1.04)	**<0.001**	2.10 (1.57)	4.72 (0.95)	1.60 (1.05)	**<0.001**
Outcomes								
Mortality 90 days	178 (4.34%)	83 (8.17%)	95 (3.08%)	**<0.001**	130 (5.79%)	54 (10.04%)	76 (4.45%)	**<0.001**
Length of stay	7.12 (8.19)	8.69 (9.26)	6.61 (7.74)	**<0.001**	7.95 (9.00)	9.91 (10.13)	7.33 (8.53)	**<0.001**
Dependency (mRS) 90 days	–	–	–	–	2.51 (2.02)	3.04 (2.05)	2.35 (1.98)	**<0.001**
Dependency 90 days (mRS ≥3)	–	–	–	–	1,021 (45.5%)	307 (57.06%)	714 (41.85%)	**<0.001**

Baseline characteristics for the full cohort and the sub-cohort. Values in mean (standard deviation) or *N* (% within AL group). *p*-value from student *t*-tests/chi square tests between high AL and moderate-low AL. Bold indicates statistically significant *p*-values. ^m^, missing data. LOS, length of stay. mRS, modified Rankin Scale; AL, allostatic load; M, mean; SD, standard deviation; ADI, area deprivation index; AF, atrial fibrillation; Diabetes m., diabetes mellitus; Diabetes m. w compl., diabetes mellitus with complications; NIHSS, National Institutes of Health Stroke Scale; IVTPA, Tissue plasminogen activator; EVT, endovascular thrombectomy; BMI, body mass index; HbA1c, hemoglobin A1c; HDL, high density lipoprotein; CRP, C-reactive protein; WBC, white blood cell count; HPF, high-power field; k/uL, kilo/microliter; SBP, systolic blood pressure; DBP, diastolic blood pressure.

Racial differences, illegal drug use, and anticoagulation reversal therapy were no longer significantly associated with high AL in the sub-cohort.

Moderate-low AL, as compared to high AL, was associated with being a woman (51.73% vs. 47.93%, *p* = 0.036), hypercholesterolemia (18.30% vs. 14.37%, *p* = 0.004), AF (25.96% vs. 20.24%, *p* < 0.001), being on anticoagulant therapy (29.77% vs. 24.02%, *p* < 0.001), IVTPA (23% vs. 16.93%, *p* < 0.001), and EVT (12.60% vs. 10.14%, *p* = 0.036).

Alcohol use went from not significant to significantly associated with moderate-low AL as compared to high AL (49.76% vs. 44.30%, *p* = 0.029) in the sub-cohort. Anticoagulant therapy and EVT were no longer significantly associated with moderate-low AL in the sub-cohort.

**Figure 1:**
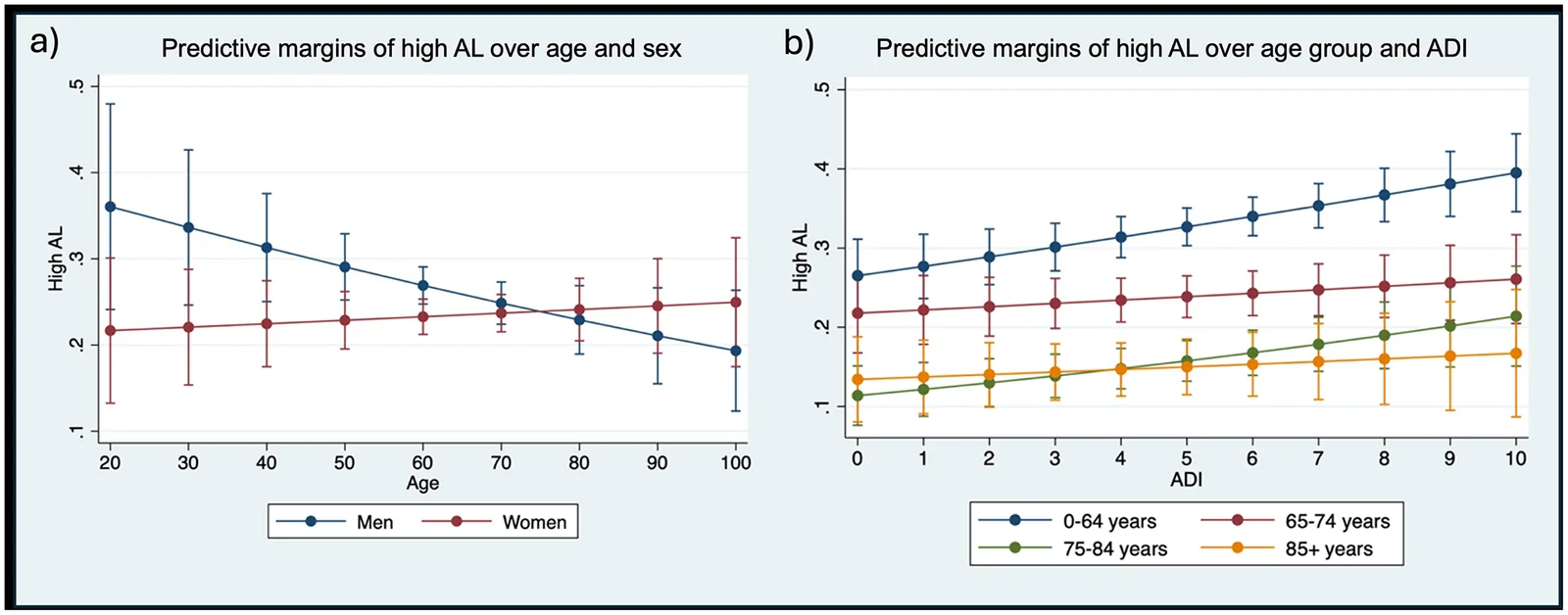
Predictive margins of high allostatic load (AL) over **(a)** age and sex, and **(b)** age group and area deprivation index score.

The logistic regression model (Supplementary Table 1) revealed that high AL was significantly associated with Age (Figure 1a) group 65-74 [odds ratio (OR) = 0.64, 95% confidence interval (CI) (0.54 to 0.78), *p* < 0.001], 75–84 [OR = 0.39, 95% CI (0.31 to 0.49), *p* < 0.001], and 85+ [OR = 0.38, 95% CI (0.28 to 0.52), *p* < 0.001], ADI (Figure 1b; OR = 1.05, 95% CI [1.02 to 1.08], *p* < 0.001), and alcohol use [OR = 0.79, 95% CI (0.67 to 0.92), *p* = 0.003]. Being a woman was close to reaching significance [OR = 0.87, 95% CI (0.74 to 1.02), *p* = 0.084].

### 3.2 Outcomes

**Figure 2:**
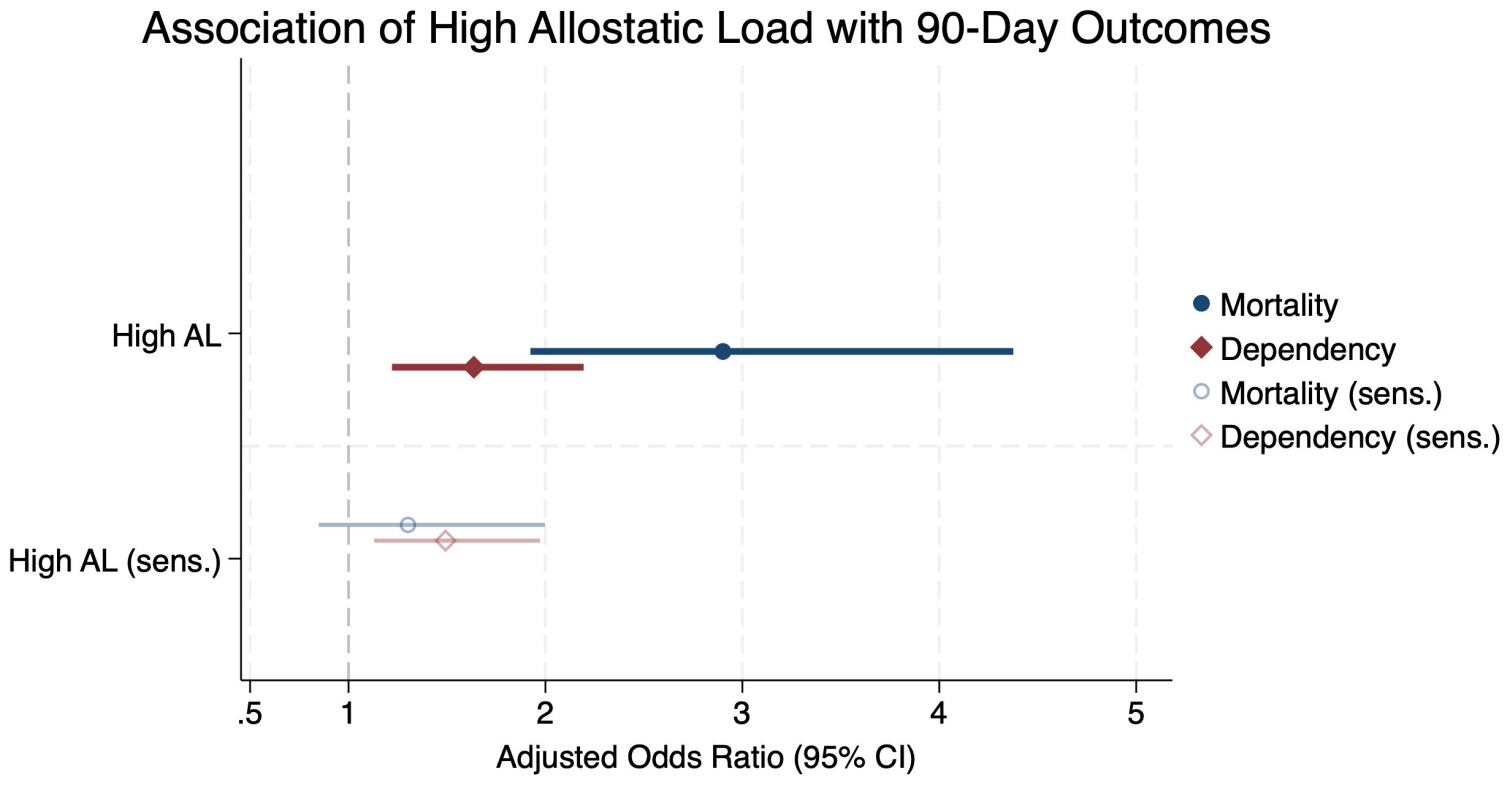
The effect of high AL across outcomes. Associations between high allostatic load and 90-day mortality and functional dependency. Points represent adjusted odds ratios (ORs), and horizontal lines indicate 95% confidence intervals. Dark symbols represent the primary allostatic load definition, and lighter symbols represent the sensitivity analysis excluding C-reactive protein and white blood cell count. Models were adjusted for age, sex, NIHSS score, reperfusion treatment, pre-stroke dependency, diabetes mellitus, atrial fibrillation, and hypertension.

Figure 2 shows the effects of high AL on all the outcomes, also depicting the results from the sensitivity analyses.

**Length of stay:** High AL, as compared to moderate-low AL, was associated with more M (SD) days spent in the hospital [8.69 (9.26) vs. 6.61 (7.74), *p* < 0.001] in the full cohort. This association was also significant in the sub-cohort.

The univariate linear regression model revealed a significant relationship between high AL and length of stay (Coef. = 2.08, 95% CI [1.51 to 2.66], *p* < 0.001). The relationship remained significant in the partially adjusted model (Coef. = 1.40, 95% CI [0.79 to 2.01], *p* < 0.001) and the multivariate [Coef. = 0.72, 95% CI (0.14 to 1.30), *p* = 0.015] models, with all NIHSS score groups, EVT, sepsis, antihypertensive therapy, anticoagulant therapy, and anticoagulation reversal therapy being significant prolonging factors, and with “black” and “other” race being significant shortening factors.

**Mortality:** High AL, as compared to moderate-low AL, was associated with higher mortality at 90 days (8.17% vs. 3.08%, *p* < 0.001) in the full cohort. This association was also significant in the sub-cohort.

The univariate logistic regression model showed a significant relationship between high AL and mortality at 90 days [OR = 2.80, 95% CI (2.07 to 3.80), *p* < 0.001]. The relationship remained significant in both the partially adjusted model [OR = 3.11, 95% CI (2.10 to 4.61), *p* < 0.001] and the multivariate [OR = 2.90, 95% CI (1.92 to 4.38), *p* < 0.001] models, with all age groups, “moderate to severe” and “severe” NIHSS score, EVT, and sepsis being significant contributing factors, and with statin therapy, anticoagulant therapy, and antiplatelet therapy being significant protective factors.

**Dependency:** High AL, as compared to moderate-low AL, was associated with higher mRS at 90 days [3.04 (2.05) vs. 2.35 (1.98), *p* < 0.001] in the sub-cohort.

The univariate logistic regression model revealed a significant relationship between high AL and dependency [OR = 1.85, 95% CI (1.52 to 2.25), *p* < 0.001]. The relationship remained significant in both the partially adjusted model [OR = 1.75, 95% CI (1.32 to 2.32), *p* < 0.001] and the multivariate [OR = 1.64, 95% CI (1.22 to 2.19), *p* < 0.001] models, with all age groups, all NIHSS score groups, sepsis, antihypertensive therapy, and anticoagulation reversal therapy being significant contributing factors, and with IVTPA being a significant protective factor. Anticoagulant therapy, statin therapy, and antiplatelet therapy approached significance as protective factors.

**Sensitivity analyses:** The sensitivity analyses excluding CRP and WBC produced outcome-specific effects (Supplementary Tables 2–4). The association between high AL and 90-day mortality was no longer statistically significant in either the partially adjusted models or multivariate models (all *p* > 0.10). In contrast, results for functional dependency were essentially unchanged: high AL remained a significant predictor, with effect estimates comparable to those of the primary analysis. For length of stay, the association with high AL persisted in the partially adjusted model but attenuated in the fully adjusted model (*p* = 0.081). Overall, removing inflammatory biomarkers eliminated the association between AL and 90-day mortality, whereas associations with functional outcome and length of stay were largely preserved.

## 4 Discussion

The current study investigated the association between high AL and post-stroke outcomes in AIS patients. We found that high AL was associated with overall worse outcome; longer stay at the hospital, and a higher risk of mortality and dependency 90 days after stroke. In the multivariable regression model, younger age, Black race, higher ADI, single status, illegal drug use, diabetes mellitus, sepsis, higher CCI, anticoagulation reversal therapy, antihypertensive therapy, and higher NIHSS at 6 and 24 h post stroke were associated with high AL, whereas moderate-low AL was associated with being a woman, hypercholesterolemia, AF, anticoagulant therapy, IVTPA, and EVT. Our findings are broadly consistent with previous studies demonstrating associations between AL and cardiovascular outcomes, including cardiovascular mortality, cardiovascular disease, and stroke mortality. The present findings extend this literature by demonstrating associations not only with mortality but also with stroke severity, length of hospitalization, and post-stroke dependency.

To the authors' knowledge, no previous studies have examined the association between AL and length of hospitalization among stroke patients. However, longer hospitalization in stroke patients is associated with younger age, being a man, receiving reperfusion therapy, higher dependency, and lower rate of in-hospital mortality (Lin et al., 2022). Also, high AL has been found linked to longer hospitalization and more days in the ICU in COVID-19 patients (García et al., 2025), suggesting that AL may also be relevant to hospitalization in other acute conditions, such as AIS.

High AL has previously been found linked to greater all-cause and cardiovascular mortality (Parker et al., 2022), and specifically also with greater stroke mortality (Johnson et al., 2025). However, in the latter study, this association was no longer observed after adjusting for age. The authors suggested this attenuation may be due to associations between AL and non-stroke conditions, such as inflammatory disorders, which are more common in younger populations. The current study did find an association between AL and mortality that did remain significant after adjustment for age. However, age was unexpectedly inversely related to AL in this cohort. This finding is however likely to reflect a survivorship effect or a selection bias, with low study participation among older adults with high AL.

High AL was associated with dependency at 90 days post stroke. The link between AL and post stroke dependency has not been specifically examined previously, however AL has been found predictive of disability as measured using mRS in the general population (Guidi et al., 2021). AL is also linked to disability as measured using I-ADL (instrumental activities of daily living) in older adults (Zhao et al., 2024), as well as a greater health burden for cancer patients with mobility limitations (Hollar, 2022).

In the sensitivity analyses excluding CRP and WBC, the association between AL and 90-day mortality disappeared. This suggests that the inflammatory components of the AL score contributed to the observed association with short-term mortality. Because these biomarkers may be influenced by the acute stroke itself, this finding raises the possibility that part of the observed mortality association reflects acute physiological responses to stroke rather than solely pre-existing physiological burden. By contrast, the association between high AL and dependency remained essentially unchanged, suggesting that the association between AL and functional outcome was less sensitive to the exclusion of these inflammatory biomarkers. Length of stay showed a modest attenuation, consistent with its dependence on both acute physiological burden and underlying comorbidity. Overall, these findings suggest that the prognostic associations of AL may reflect contributions from both chronic physiological dysregulation and acute inflammatory responses. However, because removing CRP and WBC also altered the composition of the AL score, the sensitivity analyses do not allow the specific biological contribution of these biomarkers to be determined.

The regression models revealed that high AL was associated with higher ADI, lower age, and not drinking alcohol. High ADI (as a measure of low SES) has previously been found linked to high AL in the general population (Kezios et al., 2022; Ribeiro et al., 2018), in cancer patients (Shen et al., 2022; Sangaramoorthy et al., 2025) and multiple sclerosis patients (O'Neill et al., 2024). ADI is linked to stroke severity (Sari et al., 2023), and stroke incident and mortality (Belau et al., 2023), in part due to delayed time to hospital (Forman et al., 2024). No studies have previously looked at the link between ADI and AL in stroke patients.

Low alcohol consumption was linked to high AL. Excessive drinking has been found associated with high AL (Memiah et al., 2022). However, being a current drinker, either light, moderate, or heavy, has also been found beneficial in comparison to lifelong abstainers or former light drinkers (Goldwater et al., 2019). It has also been found that moderate drinkers have lower AL than heavy drinking men or abstaining women (Petrovic et al., 2016). More frequent alcohol use has also been found linked to lower AL 10 years down the line (Milad and Bogg, 2020). These somewhat diverging findings indicate that the relationship between AL and alcohol is complex and not fully understood.

To the authors knowledge, this is the first study to look at the link between AL and stroke severity. We found that higher AL was linked to a higher NIHSS both 6 and 24 h after stroke. Our findings may be interpreted in light of the biological components that constitute our AL index. In this study, AL was primarily based on metabolic, inflammatory, and hemodynamic markers, including blood pressure, glycemia, lipid levels, and CRP, rather than neuroendocrine stress markers. Elevated AL in this context may therefore reflect chronic cardiometabolic and inflammatory dysregulation. Such long-standing vascular and systemic burden could contribute to reduced cerebrovascular reserve, impaired circulation, and diminished ischemic tolerance (de la Riva et al., 2024; Rizk et al., 2019). Consequently, individuals with higher AL may be predisposed to larger infarcts and more severe neurological deficits, as reflected by higher NIHSS scores. Although our AL index does not directly include neuroendocrine stress markers, it may partly capture the downstream physiological consequences of chronic psychological stress exposure. Other studies have found that more psychological aspects of long-term stress, such as perceived stress, stressful life events and an inability to cope all raise the risk for stroke (Reddin et al., 2022; Prasad et al., 2020; Aalbaek et al., 2017; Khalifa et al., 2025). Long-term stress has also been found to increase the risk for cerebrovascular disease and other risk factors associated with more severe strokes (Khalifa et al., 2025). An additional consideration is the role of stroke severity in the relationship between AL and stroke severity. Although higher AL was associated with higher NIHSS scores at both 6 and 24 h, several components of the AL index were measured during the acute hospitalization and may themselves have been influenced by the stroke. Consequently, the observed association should not be interpreted as establishing a causal relationship between AL and stroke severity. Rather, the persistence of significant associations after adjustment for NIHSS indicates that the association between AL and post-stroke outcomes was not fully explained by stroke severity. Future studies incorporating pre-stroke measures of AL are needed to better clarify the temporal and causal relationships between AL, stroke severity, and clinical outcomes.

These findings suggest that AL captures cumulative physiological burden relevant to stroke recovery and long-term function, beyond traditional vascular risk factors. As a composite marker involving multiple biological systems, AL may provide complementary information for risk stratification and outcome prediction in acute ischemic stroke, particularly for functional outcomes. Importantly, this potential value lies in integrating AL into existing clinical frameworks rather than replacing established prognostic indicators. Beyond serving as a general marker of accumulated biological burden, AL may have potential clinical utility in guiding decision making after stroke. If higher AL reflects reduced physiological reserve and increased vulnerability, it could help identify patients at risk of more severe neurological deficits and potentially poorer early recovery. If validated in future studies, AL could potentially help identify patients who may benefit from closer monitoring and more individualized post-stroke care. However, given the observational nature of the present study, these potential clinical applications remain speculative and require prospective validation. In this way, AL may represent a promising candidate marker for individualized risk assessment following stroke, although its role in clinical decision making remains to be established.

The large sample size and the inclusion of both in-hospital and 90-day outcomes strengthen the robustness of the findings, and, to our knowledge, this is the first study to examine AL in relation to stroke severity and post-stroke functional outcomes. Several limitations should be considered. First, our AL index did not include neuroendocrine measures, which were central to the original conceptualization of AL (McEwen and Stellar, 1993). Consequently, it primarily reflects metabolic, cardiovascular, and inflammatory dysregulation and should be interpreted as a marker of multisystem physiological burden rather than a comprehensive assessment of AL in its original sense. Nevertheless, similar operationalizations are common in epidemiological research and have consistently been associated with adverse health outcomes.

Second, complete-case analyses were used, meaning that participants with missing data were excluded from the multivariable models. If missingness was associated with participant characteristics or outcomes, selection bias may have been introduced, potentially affecting the generalizability of the findings. Also, the exact analytical sample size for each fully adjusted regression model could not be reported. This limits transparency regarding the number of participants included in individual analyses. In addition, approximately half of the study population had available 90-day mRS data. Because participants with and without follow-up data could not be compared, the potential for selection bias due to loss to follow-up cannot be excluded.

Third, the threshold used to define high AL (≥ 4) was based on previous literature rather than being derived from the distribution of the present cohort. Furthermore, sensitivity analyses using alternative parameterizations of AL (e.g., continuous values or quartiles) were not performed. Consequently, the robustness of the observed associations across different definitions of AL remains to be established.

Finally, although AL was independently associated with stroke outcomes, the present study did not evaluate whether AL provides incremental prognostic value beyond established predictors such as age and NIHSS using discrimination metrics (e.g., AUC or C-statistics). Therefore, the clinical utility of incorporating AL into existing prognostic models remains uncertain. Future studies should evaluate the incremental prognostic value of AL, examine alternative approaches to modeling AL, and explore whether interventions targeting chronic stress and multisystem dysregulation may improve post-stroke recovery.

## 5 Conclusion

High AL may represent a reduced physiological reserve and multisystem dysregulation that increases vulnerability to poor recovery after stroke. These findings suggest that AL may serve as a clinically accessible marker of biological resilience and may help identify patients at increased risk of poor post-stroke outcomes.

## Data Availability

The datasets presented in this article are not readily available because the dataset generated and analyzed during the current study is not publicly available due to patient privacy regulations and risk of identification but are available on reasonable request, subject to approval by the relevant ethics committee and data protection regulations. Requests to access the datasets should be directed to Farhaan Vahidy, Farhaan.Vahidy@memorialhermann.org.
