# Supplementary material for: Higher allostatic load at hospitalization is linked to worse outcome 90 days after stroke
Source: Front Stroke. 2026 Aug 14;5:1847419. doi: 10.3389/fstro.2026.1847419 (PMC13521850; doi:10.3389/fstro.2026.1847419)
Supplement: Supplementary file 1 [file Table_1.docx]

***Higher Allostatic Load at Hospitalization is Linked to Worse Outcome 90 Days after Stroke***

**SUPPLEMENTARY MATERIAL**

**Supplementary table 1.** Factors associated with high allostatic load at hospital admission: multivariable logistic regression analysis.

|  | **High AL** | | |
| --- | --- | --- | --- |
| **Variable** | **OR** | **95% CI** | ***p*-value** |
| Age group (ref: 0-64) |  |  |  |
| 65-74 | .64 | .54 to .78 | ***p* < .001** |
| 75-84 | .39 | .31 to .49 | ***p* < .001** |
| 85 + | .38 | .28 to .52 | ***p* < .001** |
| Female sex | .87 | .74 to 1.02 | *p* = .084 |
| Marital status (ref: married/partner) |  |  |  |
| Divorced, separated, or widowed | .95 | .77 to 1.17 | *p* = .64 |
| Single | 1.08 | .90 to 1.31 | *p* = .39 |
| Race (ref: White) |  |  |  |
| Asian | .88 | .61 to 1.28 | *p* = .50 |
| Black | 1.03 | .87 to 1.23 | *p* = .73 |
| Other | .92 | .61 to 1.39 | *p* = .68 |
| ADI state | 1.05 | 1.02 to 1.08 | ***p* < .001** |
| Tobacco use | 1.01 | .86 to 1.19 | *p* = .90 |
| Alcohol use | .79 | .67 to .92 | ***p* = .003** |
| Illegal drug use | 1.09 | .84 to 1.41 | *p* = .53 |

**Supp. table. 1:** *Odds ratios (ORs), 95% confidence intervals (CIs), and p-values are from a multivariable logistic regression model with high allostatic load (AL) as the dependent variable. Odds ratios >1 indicate increased odds of belonging to the high AL group. The reference categories were age 0–64 years, male sex, married/partner, and White race. ADI = Area Deprivation Index.*

**Supplementary table 2.** Sensitivity analysis: Linear regression for length of stay

|  | **Partially adjusted** | | | **Multivariate** | | |
| --- | --- | --- | --- | --- | --- | --- |
|  | Coef. | 95% CI | *p*-value | Coef. | 95% CI | *p*-value |
| High AL | .85 | .28 to 1.43 | **.004** | .48 | -.06 to 1.03 | .081 |
| Age group (ref: 0-64) |  |  |  |  |  |  |
| 65-74 | .56 | -.09 to 1.21 | .091 | .15 | -.47 to .77 | .63 |
| 75-84 | .45 | -.26 to 1.17 | .22 | -.04 | -.73 to .65 | .90 |
| 85+ | -.49 | -1.38 to .40 | .28 | -.85 | -1.70 to .01 | .050 |
| Female sex | -.37 | -.89 to .15 | .16 | -.28 | -.77 to .21 | .26 |
| Race (ref: White) |  |  |  |  |  |  |
| Asian | -.04 | -1.31 to 1.22 | .95 | .06 | -1.13 to 1.24 | .93 |
| Black | -1.76 | -3.50 to -.02 | **.048** | -1.85 | -3.48 to -.22 | **.026** |
| Other | -1.67 | -2.89 to -.44 | **.008** | -1.56 | -2.71 to -.42 | **.008** |
| NIHSS (ref: 0) |  |  |  |  |  |  |
| Minor | 1.17 | .32 to 2.02 | **.007** | 1.01 | .21 to 1.80 | **.013** |
| Moderate | 4.97 | 4.05 to 5.89 | **< .001** | 4.12 | 3.26 to 4.99 | **< .001** |
| Moderate to severe | 11.23 | 9.89 to 12.58 | **< .001** | 9.78 | 8.51 to 11.05 | **< .001** |
| Severe | 9.60 | 8.30 to 10.91 | **< .001** | 7.51 | 6.26 to 8.76 | **< .001** |
| IV tPA | .24 | -.41 to .89 | .47 | .06 | -.55 to .67 | .85 |
| EVT | 2.35 | 1.49 to 3.21 | **< .001** | 1.98 | 1.17 to 2.79 | **< .001** |
| Hypercholesterolemia |  |  |  | -.13 | -.79 to .53 | .70 |
| Sepsis |  |  |  | 8.15 | 7.05 to 9.25 | **< .001** |
| Statin therapy |  |  |  | .01 | -.76 to .76 | .99 |
| Antihypertensive therapy |  |  |  | 1.53 | .83 to 2.22 | **< .001** |
| Anticoagulant therapy |  |  |  | .71 | .14 to 1.27 | **.015** |
| Anticoagulation reversal therapy |  |  |  | 11.37 | 9.55 to 13.18 | **< .001** |
| Antiplatelet therapy |  |  |  | -.56 | -1.25 to .12 | .11 |

Supp.Table 2*: Results from linear regression models using length of stay as the dependent variable. The partially adjusted model included age, sex, race, NIHSS, IV tPA, and EVT. The multivariable model additionally included hypercholesterolemia, sepsis, statin therapy, antihypertensive therapy, anticoagulant therapy, anticoagulation reversal therapy, and antiplatelet therapy. Bold p-values indicate statistical significance (p < .05).* ***Abbreviations*** *Coef. = regression coefficient, CI = confidence interval, AL = allostatic load, NIHSS = National Institutes of Health Stroke Scale, IV tPA = intravenous tissue plasminogen activator, EVT = endovascular thrombectomy.*

**Supplementary table 3.** Sensitivity analysis: Linear regression for mortality

|  | OR | 95% CI | p-value | OR | 95% CI | p-value |
| --- | --- | --- | --- | --- | --- | --- |
| High AL | 1.33 | .88 to 2.01 | .17 | 1.30 | .85 to 2.00 | .23 |
| Age group (ref: 0-64) |  |  |  |  |  |  |
| 65-74 | 1.63 | .98 to 2.69 | .058 | 2.40 | 1.40 to 4.11 | **.001** |
| 75-84 | 2.04 | 1.20 to 3.48 | **.009** | 2.96 | 1.66 to 5.25 | **< .001** |
| 85+ | 2.39 | 1.37 to 4.18 | **.002** | 3.62 | 1.97 to 6.64 | **< .001** |
| Female sex | .88 | .61 to 1.28 | .50 | .89 | .60 to 1.32 | .56 |
| Race (ref: White) |  |  |  |  |  |  |
| Asian | .66 | .29 to 1.51 | .32 | .82 | .34 to 1.95 | .66 |
| Black | 1.30 | .46 to 3.71 | .62 | 1.42 | .47 to 4.24 | .53 |
| Other | 1.13 | .52 to 2.47 | .75 | 1.21 | .54 to 2.72 | .65 |
| NIHSS (ref: 0) |  |  |  |  |  |  |
| Minor | 1.27 | .36 to 4.42 | .71 | 1.20 | .34 to 4.19 | .78 |
| Moderate | 4.25 | 1.28 to 14.06 | **.018** | 3.14 | .94 to 10.52 | .064 |
| Moderate to severe | 14.79 | 4.29 to 50.98 | **< .001** | 9.37 | 2.67 to 32.94 | **< .001** |
| Severe | 36.19 | 10.88 to 120.36 | **< .001** | 19.10 | 5.62 to 64.97 | **< .001** |
| IV tPA | 1.04 | .68 to 1.59 | .86 | .95 | .61 to 1.48 | .83 |
| EVT | 1.76 | 1.16 to 2.68 | **.008** | 1.76 | 1.13 to 2.73 | **.012** |
| Hypercholesterolemia |  |  |  | .96 | .54 to 1.71 | .90 |
| Sepsis |  |  |  | 3.04 | 1.86 to 4.98 | **< .001** |
| Statin therapy |  |  |  | .47 | .29 to .76 | **.002** |
| Antihypertensive therapy |  |  |  | .86 | .51 to 1.44 | .55 |
| Anticoagulant therapy |  |  |  | .56 | .35 to .90 | **.018** |
| Anticoagulation reversal therapy |  |  |  | 1.47 | .64 to 3.41 | .36 |
| Antiplatelet therapy |  |  |  | .57 | .37 to .90 | **.015** |

**Supp.table.3::** *Results are from logistic regression models. The partially adjusted and multivariable models included the same covariates as in the primary analyses. Bold p-values indicate statistical significance (p < .05).* ***Abbreviations:*** *OR = odds ratio, CI = confidence interval, AL = allostatic load, NIHSS = National Institutes of Health Stroke Scale, IV tPA = intravenous tissue plasminogen activator, EVT = endovascular thrombectomy.*

**Supplementary table 4.** Sensitivity analysis: Linear regression for dependency

|  | **Partially adjusted** | | | | **Multivariate** | | |  |
| --- | --- | --- | --- | --- | --- | --- | --- | --- |
| Variable | OR | 95% CI | *p*-value | OR | | 95% CI | *p-*value | |
| High AL | 1.53 | 1.17 to 2.00 | **.002** | 1.49 | | 1.13 to 1.97 | **.005** | |
| Age group (ref: 0-64) |  |  |  |  | |  |  | |
| 65-74 | 1.36 | 1.01 to 1.83 | **.043** | 1.37 | | 1.01 to 1.87 | **.046** | |
| 75-84 | 2.40 | 1.72 to 3.36 | **< .001** | 2.47 | | 1.74 to 3.52 | **< .001** | |
| 85+ | 4.21 | 2.80 to 6.34 | **< .001** | 4.48 | | 2.92 to 6.87 | **< .001** | |
| Female sex | 1.14 | .90 to 1.45 | .29 | 1.12 | | .88 to 1.43 | .37 | |
| Race (ref: White) |  |  |  |  | |  |  | |
| Asian | 1.13 | .62 to 2.09 | .69 | 1.25 | | .66 to 2.38 | .49 | |
| Black | .87 | .38 to 2.00 | .74 | .88 | | .37 to 2.08 | .77 | |
| Other | .84 | .47 to 1.53 | .57 | .89 | | .47 to 1.66 | .71 | |
| NIHSS (ref: 0) |  |  |  |  | |  |  | |
| Minor | 2.52 | 1.53 to 4.15 | **< .001** | 2.38 | | 1.43 to 3.94 | **.001** | |
| Moderate | 18.00 | 10.73 to 30.19 | **< .001** | 16.07 | | 9.52 to 27.13 | **< .001** | |
| Moderate to severe | 97.00 | 46.61 to 201.87 | **< .001** | 82.96 | | 39.32 to 175.04 | **< .001** | |
| Severe | 424.97 | 136.82 to 1319.99 | **< .001** | 313.45 | | 100.06 to 981.90 | **< .001** | |
| IV tPA | .46 | .35 to .62 | **< .001** | .42 | | .31 to .56 | **< .001** | |
| EVT | .78 | .55 to 1.10 | .15 | .76 | | .53 to 1.10 | .15 | |
| Hypercholesterolemia |  |  |  | 1.29 | | .92 to 1.81 | .14 | |
| Sepsis |  |  |  | 8.47 | | 3.61 to 19.90 | **< .001** | |
| Statin therapy |  |  |  | .69 | | .47 to 1.01 | .057 | |
| Antihypertensive therapy |  |  |  | 1.51 | | 1.03 to 2.22 | **.034** | |
| Anticoagulant therapy |  |  |  | .77 | | .58 to 1.02 | .065 | |
| Anticoagulation reversal therapy |  |  |  | 6.42 | | 2.02 to 20.41 | **.002** | |
| Antiplatelet therapy |  |  |  | .73 | | .52 to 1.02 | .065 | |

**Supp.table.4::** *Results are from logistic regression models. The partially adjusted and multivariable models included the same covariates as in the primary analyses. Bold p-values indicate statistical significance (p < .05).* ***Abbreviations:*** *OR = odds ratio, CI = confidence interval, AL = allostatic load, NIHSS = National Institutes of Health Stroke Scale, IV tPA = intravenous tissue plasminogen activator, EVT = endovascular thrombectomy.*
